# MAS C-Terminal Tail Interacting Proteins Identified by Mass Spectrometry- Based Proteomic Approach

**DOI:** 10.1371/journal.pone.0140872

**Published:** 2015-10-20

**Authors:** Kalyan C. Tirupula, Dongmei Zhang, Appledene Osbourne, Arunachal Chatterjee, Russ Desnoyer, Belinda Willard, Sadashiva S. Karnik

**Affiliations:** 1 Department of Molecular Cardiology, Cleveland Clinic, Ohio, United States of America; 2 Proteomics Laboratory, Lerner Research Institute, Cleveland Clinic, Ohio, United States of America; 3 Cleveland Clinic Lerner College of Medicine at Case Western Reserve University, Cleveland Clinic, Ohio, United States of America; Hungarian Academy of Sciences, HUNGARY

## Abstract

Propagation of signals from G protein-coupled receptors (GPCRs) in cells is primarily mediated by protein-protein interactions. MAS is a GPCR that was initially discovered as an oncogene and is now known to play an important role in cardiovascular physiology. Current literature suggests that MAS interacts with common heterotrimeric G-proteins, but MAS interaction with proteins which might mediate G protein-independent or atypical signaling is unknown. In this study we hypothesized that MAS C-terminal tail (Ct) is a major determinant of receptor-scaffold protein interactions mediating MAS signaling. Mass-spectrometry based proteomic analysis was used to comprehensively identify the proteins that interact with MAS Ct comprising the PDZ-binding motif (PDZ-BM). We identified both PDZ and non-PDZ proteins from human embryonic kidney cell line, mouse atrial cardiomyocyte cell line and human heart tissue to interact specifically with MAS Ct. For the first time our study provides a panel of PDZ and other proteins that potentially interact with MAS with high significance. A ‘cardiac-specific finger print’ of MAS interacting PDZ proteins was identified which includes DLG1, MAGI1 and SNTA. Cell based experiments with wild-type and mutant MAS lacking the PDZ-BM validated MAS interaction with PDZ proteins DLG1 and TJP2. Bioinformatics analysis suggested well-known multi-protein scaffold complexes involved in nitric oxide signaling (NOS), cell-cell signaling of neuromuscular junctions, synapses and epithelial cells. Majority of these protein hits were predicted to be part of disease categories comprising cancers and malignant tumors. We propose a ‘MAS-signalosome’ model to stimulate further research in understanding the molecular mechanism of MAS function. Identifying hierarchy of interactions of ‘signalosome’ components with MAS will be a necessary step in future to fully understand the physiological and pathological functions of this enigmatic receptor.

## Introduction

MAS is a G protein-coupled receptor (GPCR) discovered as the product of *Mas* oncogene [1]. Over expression of MAS in heterologous cells transforms the cells through activation of both heterotrimeric and small G-proteins [2–5]. MAS is expressed in the heart, kidney, brain, testis and several other tissues [6–12]. Importance of MAS in maintaining normal cardiovascular homeostasis is gaining considerable attention [13–19]. In the heart and kidney, MAS function prevents ischemia/reperfusion injury by enhancing blood flow and minimizing infarct size. Efforts are currently underway to modulate MAS function for protective and therapeutic purposes [20–22]. Recently, we showed that MAS activates G protein signaling pathways and undergoes functional desensitization in response to non-peptide ligands [23]. However, MAS signaling was atypical in response to endogenous peptide ligands. The physiological ligand, Neuropeptide FF (NPFF) produced functional selective MAS signaling without functional desensitization. Whereas, the putative endogenous cardiovascular ligand angiotensin (1–7) potentiated an NPFF-like response of MAS only at non-physiological ligand concentration [23]. In both scenarios, a G protein independent component of signaling response of MAS was apparent. The molecular mechanism of atypical signaling, desensitization and G protein independent signaling observed in MAS is currently unknown.

The C-terminal tail (Ct) in GPCRs is known to play an important role in regulating G-protein independent functions. In several GPCRs the last four amino acids at the C-terminus constitute a PDZ-binding motif (PDZ-BM) which is known to interact with PDZ domain (domain present in postsynaptic density protein (PSD95), drosophila disc large tumor suppressor (DlgA) and zonula occludens-1 (ZO-1)) containing proteins [24]. PDZ proteins are a family of scaffolding proteins that are widely distributed in metazoans [25,26]. There are at least 250 PDZ proteins in the human proteome which regulate key cellular process involving cell polarity, inter-cellular junctions and several signal transduction pathways [27,28]. PDZ proteins have distinct tissue expression profiles and are shown to regulate signaling, trafficking and subcellular organization of supramolecular complexes including GPCRs [24,29].

In MAS, the C-terminal amino acids -ETVV- represents a class 1d PDZ-BM [30,31], which is similar to those present in other GPCRs such as frizzled-4 (ETVV), lysophosphatidic acid receptor 1 (HSVV) and sphingosine 1-phosphate receptor 2 (NTVV) which are known to interact with several PDZ proteins [32–35]. A direct interaction of MAS Ct with PDZ proteins, PSD-95 (also known as DLG4 or SAP90) and scribble that are known to engage class 1d PDZ-BM, have been previously reported suggesting the existence of a *bona fide* PDZ-BM in MAS [36,37]. These findings lead us to speculate that MAS interacts with different PDZ proteins in different cells or tissues which ultimately dictate its tissue- or cell-specific function.

In this study, we performed pull-down assays with biotin tagged MAS Ct as a bait and three independent cell lysates: (1) human embryonic kidney cell line (HEK293), (2) mouse atrial cardiomyocyte cell line (HL-1) and (3) human heart tissue as prey proteins. The proteins that were pulled-down were identified by mass spectrometry (LC-MS). Both PDZ and non-PDZ proteins were identified in different lysates to significantly interact with MAS Ct suggesting likely role of multi-protein complexes in MAS function. We validated direct and specific interaction of full-length MAS with PDZ proteins in cells using ΔPDZ-MAS mutant lacking the PDZ-BM. Ingenuity pathway analysis (IPA) of the protein hits suggests cell-cell junction signaling and cancers as the key signaling pathway and disease category, respectively.

## Materials and Methods

### Peptides, reagents and antibodies

The Ct peptide containing the 25 amino acids of human MAS receptor (amino acid residues 301 to 325) was synthesized at the Molecular Biotechnology Core at Lerner Research Institute (Cleveland Clinic, Cleveland, OH) with the following two modifications–(1) the peptide is biotinylated at N-terminus and (2) cysteine at position 316 is replaced with serine to prevent oxidation products of the peptide. The final peptide sequence is: *Biotin*-R^301^AFKDEMQPRRQKDNSNTVTV**ETVV** ^325^, wherein the amino acid numbering is in superscript, PDZ motif is shown in bold and the cysteine to serine mutation at position 316 is underlined. The peptide stocks at 10 mM concentration were dissolved in sterile Milli-Q water (Merck Millipore, Billerica, MA) and stored at -20°C.

The cell and tissue lysis buffers, Mammalian Protein Extraction Reagent buffer (M-PER) and Tissue Protein Extraction Reagent buffer (T-PER), respectively were from Thermo Scientific (Rockford, IL). Phosphatase inhibitors, NeutrAvidin agarose resin and Gel Code® Blue stain reagent were also from Thermo Scientific (Rockford, IL). Protease inhibitors, Claycomb culture medium, non-enzymatic cell dissociation solution and β-mercaptoethanol were purchased from Sigma-Aldrich (St. Louis, MO). The Benzonase® nuclease and Amicon® Ultra centrifugal filter units were purchased from Merck Millipore (Billerica, MA). Laemmli buffer (2x) was purchased from Bio-Rad (Hercules, CA). The pcDNA3.1 and pcDNA 5/TO plasmid vectors and NuPAGE Bis-Tris gels were from Life Technologies (Grand Island, NY).

The anti-c-myc (9E10) monoclonal antibody was obtained from the Hybridoma Core Facility at Lerner Research Institute (Cleveland Clinic, Cleveland, OH), while anti-MAS goat polyclonal antibody (sc-54846) was purchased from Santa Cruz Biotechnology, Inc. (Santa Cruz, CA). The mouse monoclonal antibodies for pan-MAGUK (K28/86) and GAPDH (clone 6C5) were obtained from Merck Millipore (Billerica, MA) and Life Technologies (Grand Island, NY), respectively. Anti-flotillin-1 rabbit polyclonal and anti-calnexin (C5C9) rabbit monoclonal antibodies were from Cell Signaling Technology (Danvers, MA). The species-specific secondary antibodies conjugated either with horseradish peroxidase or with near-infrared (IR) were from GE Healthcare (Buckinghamshire, UK) and LI-COR Biosciences (Lincoln, NE), respectively.

MAS-selective ligands AR234960 (AR-*agonist*) and AR244555 (AR-*inverse agonist*) were a gift from Arena Pharmaceuticals, Inc. (San Diego, CA). Neuropeptide FF (NPFF) was synthesized by the Molecular Biotechnology Core at Lerner Research Institute (Cleveland, OH).

### Cell culture and preparation of cell lysates for pull-down assays

HEK293 cells were grown in Dulbecco's modified Eagle's medium supplemented with 10% fetal bovine serum (FBS), and 100 units/ml penicillin-streptomycin (Pen-Strep). HL-1 cells were grown in claycomb medium supplemented with 4 mM L-Glutamine, 10% FBS and 100 units/ml Pen-Strep [38]. Cells were maintained in a humidified incubator at 37°C and 5% CO_2_. For the pull-down experiments, cells from two confluent T-150 flasks were washed twice with Dulbecco's Phosphate-Buffered Saline (D-PBS; 1.47 mM KH_2_PO_4_, 138 mM NaCl, 2.67 mM KCl, 8.1 mM Na_2_HPO_4_, pH 7.3) and then harvested in non-enzymatic cell dissociation solution. The dislodged cells were collected by centrifuging at 1000 g for 10 min and the cell pellets were snap-frozen in liquid nitrogen and stored at -80°C.

The frozen cell pellets from two T-150 flasks were thawed on ice and cells were lysed for 10 min with 1 ml of M-PER with 137 mM NaCl, protease inhibitors, phosphatase inhibitors and 100 U/ml Benzonase at room temperature. The solubilized cell lysate containing cytosolic cell fraction was separated by pelleting nuclei and other cell debris by centrifuging at 14,000 g for 15 min at 4°C.

### Preparation of human cardiac tissue lysates for pull-down assays

The human non-failing heart tissue samples with left ventricular hypertrophy were obtained from the Department of Cardiovascular Medicine at Cleveland Clinic (Cleveland, OH). Written informed consent from the donor or the next of kin was obtained for the use of these samples in research under Cleveland Clinic Institutional Review Board protocol #2378, which specifically approved this study. These tissue samples had relatively high expression of MAS as measured by real-time quantitative PCR analysis compared to non-failing heart tissue samples (unpublished data) and hence were included in this study. The frozen tissue sections were cut carefully into small pieces with a scalpel on dry ice. Approximately 50 mg of tissue was transferred into pre-chilled Teflon capsules with pre-chilled steel ball and 500 μl of ice-cold T-PER with protease inhibitors, phosphatase inhibitors and 10 U/ml Benzonase. The Teflon capsules were transferred to mechanical homogenizer (Mikro-Dismembrator from Sartorius) and the samples were agitated at 1500 rpm for 90 s. The homogenized samples were transferred to a pre-chilled 1.5 ml tubes and soluble tissue lysate was collected by centrifuging the samples at 10,000 g for 5 min at 4°C.

### Pull-down assay

The high capacity NeutrAvidin agarose resin was equilibrated at room temperature and 500 μl of 50% bead slurry was transferred to 1.5 ml tubes. The beaded agarose resin was washed three to four times by resuspending with 500 μl of D-PBS and centrifuging at 500 g for 1 min. The washed resin bed, approximately 250 μl, was mixed with 500 μl of 1mM biotinylated MAS Ct peptide in D-PBS and incubated for 15 min with nutation. The resin with immobilized MAS Ct was washed at least three times with 500 μl of D-PBS to remove unbound peptide. The resin alone negative controls were treated identically to the experimental samples except that the biotinylated MAS Ct peptide was not added to the negative controls. The MAS Ct bound and resin alone controls were mixed independently with 1 ml of HEK293 cell lysates (~4 mg), HL-1 cell lysates (~6 mg) or cardiac tissue lysates (~600 μg) and incubated overnight at 4°C with nutation. On the following day, the unbound cell lysate was separated by centrifuging at 500 g for 1min. The bead resin was then washed five to six times with 500 μl of D-PBS. The proteins bound specifically to the Ct and non-specifically to the resin were released without disrupting the biotin-Ct and NeutrAvidin interaction by incubating with 250 μl of D-PBS containing 2% sodium dodecyl sulfate (SDS) for 30min [39]. This step was repeated twice and the protein elutions were pooled by centrifuging at 500 g for 1min. To the protein elutions (~500 μl) 14ml of D-PBS was added to dilute the SDS and the proteins were then concentrated to a final volume of 50 μl in an Amicon® Ultra centrifugal filter unit with 3 kDa molecular weight cut-off.

Small aliquots of samples (10 μl each of input and unbound fractions; 25 μl each of wash and elution fractions) were removed at each step and were mixed with 2x Laemmli buffer and 5% β-mercaptoethanol. The samples were heat treated at 70°C to 80°C for 10 min and separated on NuPAGE Bis-Tris gels. Proteins were transferred from the gels to nitrocellulose membranes and subsequently stained with Ponceau S (Sigma-Aldrich, St. Louis, MO). The membrane was washed in water to remove the Ponceau S stain and subsequently blocked with 5% dry milk powder in TBST (20 mM Tris, 137 mM NaCl, 0.1% (v/v) Tween 20 pH 7.6). The blots were later incubated overnight at 4°C with pan-MAGUK antibody in TBST. The blots were washed with TBST and incubated with horseradish peroxidase conjugated sheep anti-mouse secondary antibody for 1h at room temperature. The blots were washed and developed with ECL Plus Western Blotting Detection system (GE Healthcare, Buckinghamshire, UK).

### Mass spectrometry (MS)

Equal amounts of elution protein samples (~10 μg) from the Ct immobilized resin ('+') or resin alone ('–') pull-down assay were mixed with 2x Laemmli buffer and 5% β-mercaptoethanol and were heat treated at 70°C to 80°C for 10 min. The protein samples were then separated by electrophoresis on NuPAGE Bis-Tris gels. The proteins in the gels were fixed with 10% acetic acid and 50% ethanol for 30 min followed by four washes with Milli-Q water. Subsequently, the gels were stained overnight with Gel Code® Blue stain Reagent according to the procedure as provided by the supplier. The gels were then submitted to the Proteomics core laboratory for MS analysis.

For the in-gel protein digestion, the bands were cut to minimize excess polyacrylamide and divided into 6 smaller pieces. The gel pieces were washed with water and dehydrated in acetonitrile. The bands were then reduced with DTT and alkylated with iodoacetamide prior to the in-gel digestion. All bands were digested in-gel using trypsin, by adding 5 μl 10 ng/μl trypsin in 50 mM ammonium bicarbonate. They were incubated overnight at room temperature to achieve complete digestion, and then extracted from the polyacrylamide in two aliquots of 30 μl 50% acetonitrile with 5% formic acid. The extracts were combined and evaporated to <10 μL in Speedvac and then suspended in 1% acetic acid to make up a final volume of ~30 μl for LC-MS analysis.

The LC-MS system was a Finnigan LTQ-Orbitrap Elite hybrid mass spectrometer system. The HPLC column was a Dionex 15 cm x 75 μm internal diameter Acclaim Pepmap C18, 2 μm, 100 Å reversed phase capillary chromatography column. Five μl volumes of the extract was injected and the peptides eluted from the column by an acetonitrile/0.1% formic acid gradient at a flow rate of 0.3 μl/min were introduced into the source of the mass spectrometer on-line. The digest was analyzed using the data dependent multitask capability of the instrument acquiring full scan mass spectra to determine peptide molecular weights and tandem mass spectra (MS/MS) to determine amino acid sequence in successive instrument scans.

Tandem mass spectra were extracted by Proteome Discoverer version 1.4.1.288. All MS/MS samples were analyzed using Mascot (Matrix Science, London, UK; version 2.3.02), X! Tandem (The GPM, thegpm.org; version CYCLONE (2010.12.01.1)) and Sequest (Thermo Fisher Scientific, San Jose, CA; version 1.4.0.288) to search either the human (HEK293 cells and cardiac tissue) or mouse (HL-1 cells) reference sequence database assuming the digestion enzyme trypsin. Searches were performed with a fragment ion mass tolerance of 0.8 Da and a parent ion tolerance of 10 PPM. Carbamidomethyl of cysteine was specified as a fixed modification and oxidation of methionine was specified as a variable modification.

For validation experiments, MS of the membrane fractions from WT and mutant MAS expressing cells was performed identically except that approximately 45 μg of samples were mixed with 4x loading buffer (1x = 2 M urea, 1% SDS, 2.5% β-mercaptoethanol with 0.005% bromophenol blue) and were separated by electrophoresis on NuPAGE Bis-Tris gels without heat treating the samples to prevent aggregation of membrane proteins. Only the regions between 75kDa to 150kDa molecular weight range were identified.

### Analysis of MS/MS based peptide and protein identifications

Scaffold (version Scaffold_4.3.4, Proteome Software Inc., Portland, OR) was used to validate MS/MS based peptide and protein identifications. Peptide identifications were accepted if they could be established at greater than 0.1% probability by the Peptide Prophet algorithm with Scaffold delta-mass correction [40]. Positive peptide identifications required Mascot ion scores greater than 40, Sequest deltaCn scores greater than 0.10 and XCorr scores greater than 1.5, 2.0, 2.2 and 2.5 for singly, doubly, triply and quadruply charged peptides, and X! Tandem required -Log(Expect Scores) scores of greater than 2.0. Protein identifications were accepted if they could be established at greater than 99.0% probability and contained at least 2 identified peptides. Protein probabilities were assigned by the Protein Prophet algorithm [41]. Proteins that contained similar peptides and could not be differentiated based on MS/MS analysis alone were grouped to satisfy the principles of parsimony.

For proteomic analysis, the relative quantity of the proteins was determined by comparing the number of spectra, termed spectral counts (SC), used to identify each protein. The numerical values used in the quantitation corresponded to the normalized spectral counts (nSC) is calculated by dividing the SC of sample by the sum of SC in the entire sample [42]. The precision of the proteomic analysis is dependent on the overall abundance of the protein having larger errors for lower abundant proteins. Due to this, proteins with SC > 10 are considered significant. The ratio of nSC of experimental sample (resin with immobilized Ct; '+') to that of back ground (resin alone controls; '–') is estimated to determine the specific binding of proteins to Ct. All protein hits described in the results are almost exclusively present in '+' sample only (nSC > 10 to ∞).

### Bioinformatics analysis

A commercial software package Ingenuity Pathway Analysis (IPA; from QIAGEN, Redwood City, CA) was used to categorize the protein hits based on the signaling network, biological function and disease involvement. All protein hits with nSC > 10 from HEK293 samples and cardiac samples (a combined a list hits from HL-1 and human cardiac tissue) were uploaded to IPA and core analysis was performed. The software identifies the biological functions most relevant to the dataset and calculates p-value using the right-tailed Fisher Exact Test. The p-values less than 0.05 indicate a statistically significant, non-random association (www.ingenuity.com).

### Cloning and expression of wild-type (WT) and mutant MAS receptors

The synthesis of WT MAS expression construct with an N-terminal *myc*-tag and its cloning into pcDNA3.1 and tetracycline/doxycycline inducible vector pcDNA 5/TO is described previously [23]. The ΔPDZ-MAS mutant was created by deleting the last five C-terminal amino acids of WT *myc*-tagged MAS in pcDNA3.1 or pcDNA 5/TO vector background using site-directed mutagenesis (Agilent Technologies, Santa Clara, CA). The sequence of ΔPDZ-MAS mutant in plasmid constructs was verified by capillary DNA sequencing at the Genomics Core at Lerner Research Institute (Cleveland Clinic, Cleveland, OH). HEK293 cells were transiently transfected with 6 μg of plasmid DNA per 100 mm cell culture plate using Fugene (Roche, Indianapolis, IN) according to the protocol provided by the supplier. Tetracycline inducible HEK293 cells stably expressing ΔPDZ-MAS were established as described previously for WT-MAS [23]. Both transiently and stably transfected cells were used for cell culture experiments.

### Membrane preparations by sucrose density floatation method

The cells transfected with WT-MAS or ΔPDZ-MAS were used for membrane preparations 60–65 h post-transfection. The cells from six 100 mm plates were washed twice with D-PBS and carefully dislodged by scraping. The cells were pelleted by centrifuging at 1000 g for 10 min. The cell pellets were re-suspended in 3 ml homogenizing buffer (HB; 20 mM Tris-HCl, 137 mM NaCl, 3 mM MgCl2, 1 mM EDTA, pH 7.5) with protease inhibitors, phosphatase inhibitors, 100 U/ml Benzonase and 50% sucrose and manually homogenized (~50 strokes) on ice in a dounce homogenizer. The homogenized cell lysates were centrifuged in a swinging bucket rotor at 20,000 g for 25–30 min at 4 C. The cell membranes that were floating on the surface were carefully collected with a wide-bore pipette tip along with the supernatant (~3 ml) and transferred to a fresh pre-chilled centrifuge tube. To this supernatant with membranes, fresh homogenizing buffer (without sucrose) was added to a final volume of 9 ml and thoroughly mixed to dilute the sucrose content from 50% to ~16% and then centrifuged at 20,000 g for 25–30 min at 4°C. The resulting supernatant (cytosolic fraction) was carefully separated into fresh tubes. The membrane pellet was thoroughly re-suspended in 500 μl of M-PER buffer with 137 mM NaCl, protease inhibitors and phosphatase inhibitors and incubated for 1h at 4°C with nutation. The M-PER dissolved membranes (Note: M-PER buffer contains CHAPS detergent to dissolve the membranes [43]) were then centrifuged at 14,000 g for 10min at 4°C. The detergent solubilized membrane supernatant was collected into a fresh tube and the insoluble membrane pellet was re-suspended in fresh 500 μl of M-PER buffer.

### Detection of MAS and other proteins in membrane and non-membrane fractions

Equal quantities of membrane and non-membrane fractions (~30 μg) were mixed with 2x Laemmli buffer and 5% β-mercaptoethanol, (Note: Heat treatment of samples was avoided to prevent aggregation of MAS which is a transmembrane protein) and separated by electrophoresis on NuPAGE Bis-Tris gels and transferred onto nitrocellulose blots for western blot analysis. The western blots were probed with c-myc, MAS, pan-MAGUK, flotillin, calnexin and GAPDH primary antibodies and suitable near-infrared (IR) dye conjugated secondary antibodies. Same blots were probed multiple times with different antibodies by stripping them with OneMinute® Advance Western Blot Stripping Buffer (GM Biosciences, Fredrick, MD). The protein bands on the blots were quantified by measuring the fluorescence on Odyssey® Infrared Imaging System (LI-COR Biosciences, Lincoln, NE) and the relative abundance of proteins was expressed as a ratio or fold increase over suitable controls.

## Results

### Pull-down of MAS Ct interacting proteins

**Fig 1:**
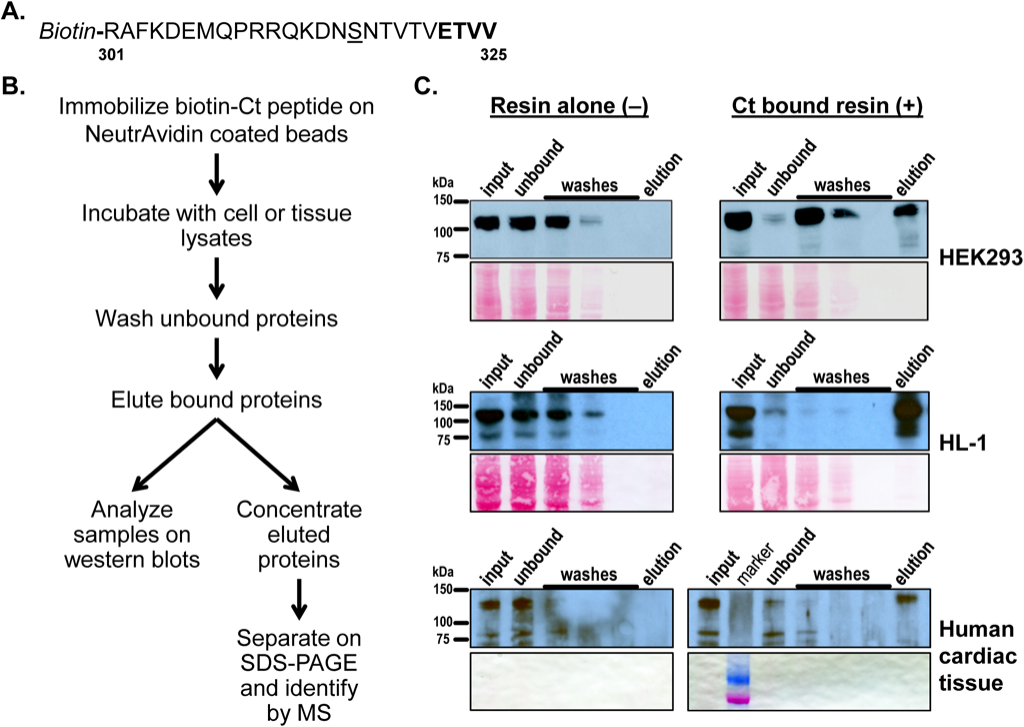
Isolating MAS Ct interacting proteins by pull down assays. **(A)** MAS Ct comprising the last 25 amino acids with an N-terminal biotin was synthesized as bait for the pull down assay. The PDZ binding motif (-E-T-V-V) in MAS is underlined while the cysteine to serine mutation in the peptide is underlined. **(B)** The major steps in the pull down assay are shown in a flow chart. **(C)** Western blot analysis of various fractions (input, unbound, washes and elutions) from pull down experiments of HEK293, HL-1 and cardiac tissue lysates. Presence of PDZ protein is detected by pan-MAGUK antibody. Below the western blots, Ponceau S stained membrane portions of corresponding regions (75 kDa to 150 kDa) from the blots are shown as a loading control.

The pull-down assays were performed with N-biotinylated MAS Ct containing the last 25 amino acids of the receptor including the PDZ motif (Fig 1A). The biotinylated Ct peptide was immobilized on NeutrAvidin agarose resin and Ct interacting proteins were isolated from HEK293, HL-1 and cardiac tissue lysates (Fig 1B; also see methods). To determine the success of the pull-down assay the samples were first analyzed on a western blot with a pan-MAGUK antibody that detects multiple proteins belonging to the membrane-associated guanylate kinases (MAGUK) sub-family of PDZ proteins. As expected, there were no signals for pan-MAGUK in the elution fractions while there was positive signal in the input, unbound and wash fractions of the resin alone control ('–') (Fig 1C). On the other hand the elution fractions in the Ct peptide bound resin ('+') samples were strongly positive for the presence of PDZ proteins. Furthermore, in the '+' samples the loss of PDZ proteins was minimal in the wash fractions compared to the input lanes suggesting specific binding of PDZ proteins to Ct. The band intensities were weaker in the human cardiac samples compared to HEK293 and HL-1 as comparatively less quantity of lysate (input) was used for the pull-down experiment (see methods). Only the elution fractions from both '–' and '+' samples were further analyzed for identification of specific MAS Ct interacting proteins.

### Identification of MAS Ct interacting proteins by Mass spectrometry (MS)

**Fig 2:**
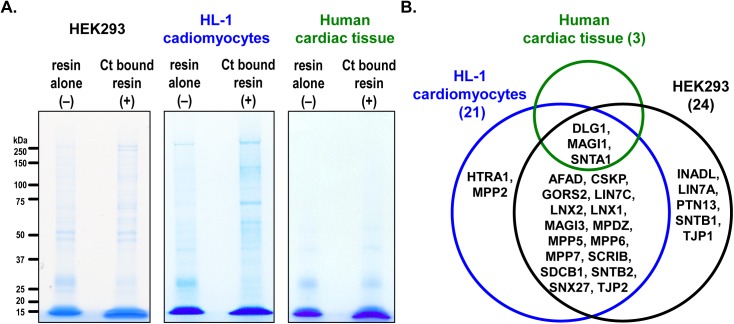
Identification of proteins from pull down assay by MS. **(A)** Protein gels for MS analysis. The elution fractions from resin alone control ('–') and Ct bound resin ('+') were resolved on SDS-PAGE gels and the entire lanes were subjected for MS analysis to identify the proteins. **(B)** Venn-diagram of PDZ proteins identified to be specifically pulled down by MAS Ct in three different sample lysates. The total numbers of PDZ proteins identified are given in parenthesis. All PDZ hits are almost exclusively present in '+' samples. List of non-PDZ proteins identified to interact with Ct are given in Table 2.

The elution fractions from the pull-down assays were concentrated and equal amounts of the proteins were separated on protein gels (Fig 2A). In these gels it is evident that there is non-specific binding of proteins in '–' controls. However, the presence of additional bands in '+' compared to '–' controls suggests the presence of specific proteins which likely interact with MAS Ct. To identify proteins pulled down in both '–' and '+', entire lanes from the gels from HEK293, HL-1 and human cardiac tissue were independently subjected to MS analysis. A total of 925, 1000 and 210 proteins were identified in HEK293, HL-1 and cardiac tissue lysates, respectively (S1, S2 and S3 Tables). Of these 24, 21 and 3 PDZ proteins were identified to specifically interact with MAS Ct in HEK293, HL-1 and cardiac tissue lysates, respectively (Fig 2B). Majority of the PDZ proteins identified were common to both HEK293 and HL-1 samples while SNTA1, DLG1 and MAGI1 were common to all three samples. The PDZ proteins identified in our screen represent a functionally diverse set of proteins as seen by the presence of different structural and functional domains such as ras association domains, serine/threonine protein kinases, arc homology 3 domain, guanylate kinase domains, Zn-finger domains, plecstrin homology domains, protein tyrosine phosphatase catalytic domain and leucine rich repeats to name a few (Table 1). Many of the PDZ proteins identified also contain multiple PDZ domains. For example, INADL (also known as PATJ) and MPDZ (also known as MUPP1) contain 10 and 13, PDZ domains, respectively.

**Table 1:** List of PDZ protein hits with their synonyms, structural and functional domains.

Uniprot name	Synonyms from IPA	Functional domains in the proteins^†^ ^,^ * ^,^ ^‡^
**AFAD**	MLLT4, AF-6, AFADIN, Gm314, I-afadin, S-AFADIN	PDZ, DIL, FHA, RA (2)
**CSKP**	CASK, CAGH39, CAMGUK, CMG, DXPri1, DXRib1, FGS4, LIN-2, MICPCH, mLin-2, MRXSNA, Pals3, TNRC8	PDZ, GuKc, L27 (2), S_TKc, SH3
**DLG1**	DLGH1, E-dlg/SAP97, hdlg, SAP-97	PDZ (3), GuKc, L27, MAGUK_N_PEST, SH3
**GORS2**	GORASP2, GOLPH2, GOLPH6, GRASP55, GRS2, p59	PDZ
**HTRA1**	ARMD7, CARASIL, HTRA, IGFBP5-protease, L56, PRSS11, PRSSS, RSPP11, SERINE PROTEASE 11/IGF binding	PDZ, IB, KAZAL
**INADL**	CIPP, hINADL, Inadl2, InaD-like, PATJ	PDZ (10), L27
**LIN7A**	LIN7, lin-7 homolog A, MALS-1, Mlin-7, protein lin-7 homolog A-like, TIP-33, Veli, VELI1	PDZ, L27
**LIN7C**	lin-7 homolog C, MALS-3, VELI	PDZ, L27
**LNX1**	E3 ubiquitin protein ligase, LNX, MPDZ, PDZRN2	PDZ (4), RING
**LNX2**	PDZRN1	PDZ (4), RING
**MAGI1**	AIP-3, BAIAP1, BAP-1, Gukmi1, Magi1d, TNRC19, WWP3	PDZ (6), GuKc, WW (2)
**MAGI3**	Slipr	PDZ (6), GuKc, WW (2)
**MPDZ**	INAD, MUPP1	PDZ (13), L27
**MPP2**	DLG2, Dlgh2, Pals4	PDZ, GuKc, L27 (2), SH3
**MPP5**	PALS1	PDZ, GuKc, L27 (2), SH3
**MPP6**	FIN15, PALS2, VAM-1	PDZ, GuKc, L27 (2), SH3
**MPP7**	Gm955	PDZ, GuKc, L27 (2), SH3
**PTN13**	FAP-1, hPTP1E, PNP1, ptp, PTP1E, PTP-BAS, PTP-BAS5E, PTP-BL, PTPL1, PTPLE, Ptpri, RIP, FRIED	PDZ (5), B41, FERM_C, KIND, PTPc
**SCRIB**	CRC, CRIB, CRIB1, SCRB1, SCRIB1, Vartul	PDZ (4), LRR (11)
**SDCB1**	MDA-9, ST1, syntenin-1, TACIP18, MDA9, SYCL, SDCBP	PDZ (2)
**SNTA1**	alpha SYNTROPHIN, SNT1, syntrophin α1, Syntrophin alpha1, TACIP1, α-1-syntrophin, α SYNTROPHIN	PDZ, PH
**SNTB1**	59-DAP, A1B, BSYN2, DAPA1B, SNT2, SNT2B1, TIP-43, syntrophin basic 1, syntrophin beta 1, syntrophin β1	PDZ, PH
**SNTB2**	Snt2, SNT2B2, SNT3, SNTL, syntrophin basic 2, syntrophin beta 2, syntrophin β2	PDZ, PH
**SNX27**	MRT1, MY014, SNTX27	PDZ, PX
**TJP1**	ZO-1	PDZ (3), GuKc, SH3, ZU5
**TJP2**	ZO-2	PDZ (3), GuKc, SH3

^**†**^Domain information is extracted from SMART Database [61,62]

*Number of domain repeats if more than one is given in parenthesis

^‡^Domain abbreviations—**B41**: band 4.1 homologues also known as ezrin/radixin/moesin (ERM) protein domains; **DIL:** class V myosin homology region; **FERM_c**: FERM C-terminal Plecstrin homology-like domain; **FHA:** forkhead associated; **GuKc:** guanylate kinase homologues; **IB:** insulin growth factor-binding protein homologues; **KAZAL:** kazal type serine protease inhibitors; **KIND:** kinase non-catalytic C-lobe domain; **L27:** domain in receptor targeting proteins Lin-2 and Lin-7; **LRR:** Leucine rich repeats; **MAGUK_N_PEST:** polyubiquitination (PEST) N-terminal domain; **PDZ:** domain present in PSD-95, Dlg, and ZO-1/2; **PH:** plecstrin homology domain; **PTPc:** protein tyrosine phosphatase, catalytic domain; **PX:** PhoX homologous domain, present in p47phox and p40phox, **RA:** ras association (RalGDS/AF-6) domain; **RING:** really interesting new gene Zn-finger domain; **S_TKc:** serine/threonine protein kinases, catalytic domain; **SH3:** src homology 3 domain; **WW:** domain with 2 conserved Trp (W) residues; **ZU5:** domain present in ZO-1 and Unc5-like netrin receptors

In addition to the PDZ proteins we found several non-PDZ proteins to be significantly enriched in '+' samples (Table 2). It is likely that the pull-down assays captured not only direct binary complexes of MAS Ct and PDZ proteins but also multi complexes involving both PDZ and non-PDZ proteins that are indirectly engaged with MAS Ct. A prediction of protein-protein association network using STRING database [44] highlights the direct physical and functional interactions of MAS Ct interacting proteins (S1 Fig). For example, there is clustering of PDZ proteins (SNTA1, SNTB1 and SNTB2) and non-PDZ proteins (DMD, DTNA, DTNB and UTRO) which were identified in both HEK293 and HL-1 datasets (S1 Fig). This cluster of proteins is a well-known scaffold for signaling proteins called dystrophin-associated protein complex (DAPC) [45]. Another cluster involving MPP7, DLG1, CASK and LIN7 form tripartite complexes (MPP7-DLG1-LIN7; DLG1-CASK-LIN7) that regulate cell polarity and stability of cell-cell junctions [46,47]. Similarly, TJP1 (also known as ZO-1), TJP2 (also known as ZO-2) and AFAD (also known as MLLT4) cluster was also identified that regulates assembly of cell-cell junctions [48]. Identification of these well-known scaffolding complexes increases the confidence in our experimental approach.

**Table 2:** List of non-PDZ proteins identified in the pull-down assays with different samples.

Sample lysate	Non-PDZ protein hits* (Number of proteins)
**HEK293**	CND1, CPVL, CTNL1, DDX21, **DMD, DTNA, DTNB, RL6,** RL14, RL27, RLA2, **RS18, SC24C, UTRO** (**14**)
**HL-1 cardiomyocytes**	**DMD**, **DTNA** [Q8CFR5]^**†**^, **DTNB** [Q8K0N0], CAMK2G [KCC2G], PP1A, RALY, RL4, **RL6,** RL7, RL8, **RS18,** RS25, SC23A, SC23B, **SC24C** [Q80U83], SGCD, SHOC2, SORL, **UTRO** [E9Q6R7] (**19** ^‡^)
**Human cardiac tissue**	ACACB, ACOT9, ARF1, CATD, EF1A1, GRP78, HBA, HMCN1, HS90A, IF4A2, NNTM, PRDX1, TNNI3 (**13**)

*All hits are almost exclusively present in '+' samples; Proteins in bold are hits present in HL-1 and HEK293; ^†^Uniprot names are moved into square brackets and alternate names are provided for easy reference.

^‡^Significance cut-off for spectral counts was set at greater than 20 instead of 10 (see methods) for this sample only to obtain a smaller protein sample set

### Bioinformatics analysis of MAS Ct interacting proteins

**Fig 3:**
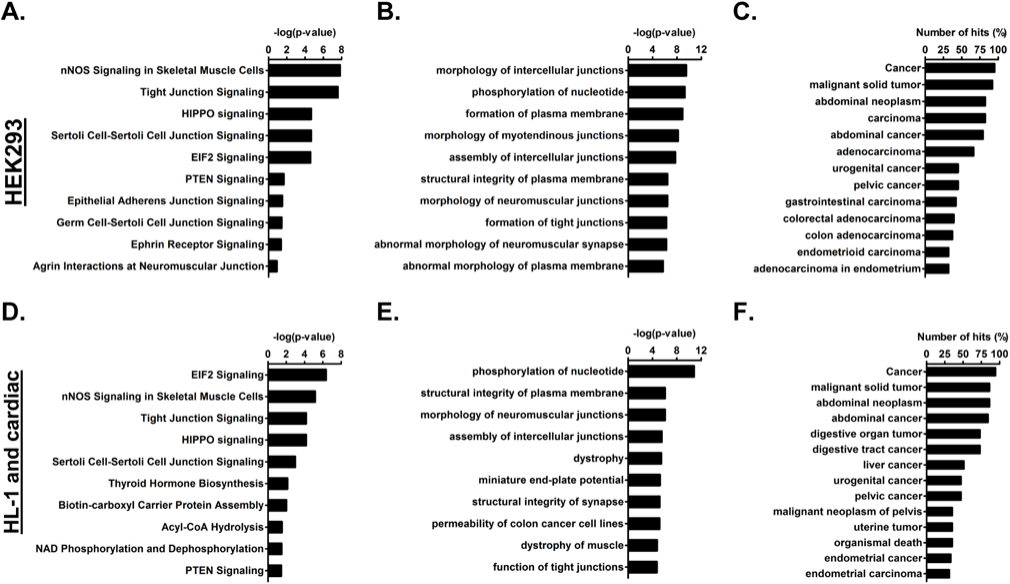
Ingenuity Pathway Analysis (IPA) of protein hits. List of IPA predicted **(A and D)** top 10 signaling networks, **(B and E)** top 10 function categories and **(C and F)** disease categories involving protein hits from **(A, B and C)** HEK293 and **(B, E and F)** HL-1 and Human cardiac tissue samples. In panels A, B, D and E a p-value of less than 0.05 (or -log(p-value) < 1.3) is considered statistically significant. In panels **C** and **F**, the numbers of protein hits that are involved in a particular disease or functional category are expressed as a percentage of total protein hits that were included in the analysis and shown as a bar graph which is truncated to show categories with greater than 30% representation.

The MAS Ct interacting proteins (both PDZ and non-PDZ) from the three samples (Fig 2B and Table 2) were uploaded into Ingenuity Pathway Analysis (IPA) software to predict the predominant signaling and functional pathways comprising these proteins. Predominantly nNOS signaling pathways, cell-cell junction, Elf2 and hippo signaling were predicted in the HEK293 sample (Fig 3A). While assembly, morphology, stabilization and function of intercellular junctions and tight junctions with specific reference to neuromuscular junctions and synapses were the predominant functional categories (Fig 3B). Interestingly, cancers of different tissues appear to be the major disease category (Fig 3C). Elf2, nNOS signaling, cell-cell junction and hippo signaling were also predicted to be major signaling networks in the cardiac samples (HL-1 and cardiac tissues; Fig 3D). Function categories similar to HEK293 hits were also predominantly predicted in cardiac samples (Fig 3E). Similar to HEK293 hits, cancers and malignant tumors are predicted to be the major disease category involving MAS Ct interacting proteins from cardiac cell and tissue lysates (Fig 3F).

### Comparison of G protein signaling in WT and ΔPDZ-MAS mutant

**Fig 4:**
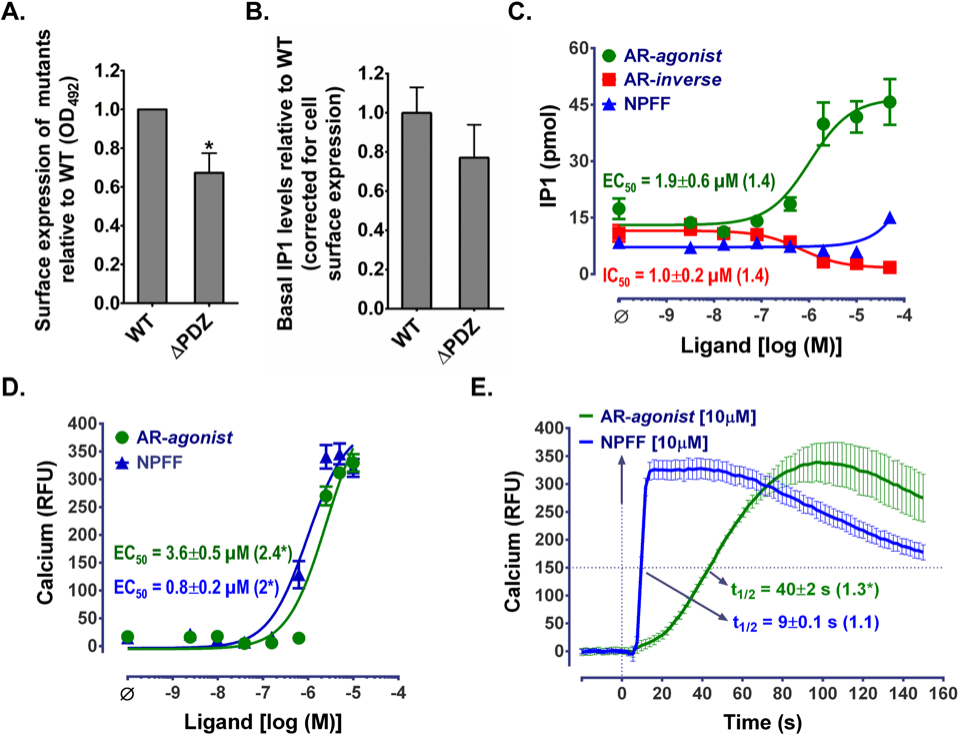
Summary of ΔPDZ-MAS expression and signaling compared to WT. **(A)** Surface expression of ΔPDZ-MAS to WT evaluated using enzyme linked immunosorbent assay (ELISA). **(B)** The bar graph showing the constitutive/basal IP1 levels (in the absence of any ligand treatment) in ΔPDZ-MAS relative to the WT after normalizing for cell surface expression. The ligand dose-responses of ΔPDZ-MAS in **(C)** IP1 and **(D)** calcium functional assays compared to WT. **(E)** The calcium flux kinetics (t_1/2_) upon treatment with NPFF and AR-agonist compared to WT. The EC_50_ or IC_50_ values are also shown in panels C and D while t_1/2_ values are shown in panel E. The relative EC_50_, IC_50_ and t_1/2_ values as compared to WT (fold increase) are shown in parentheses. Data for WT and the experimental methodology for ELISA, IP1 and calcium functional assays have been reported previously [23]; Values are mean±SEM from at least two independent experiments; Statistical significance (t-test)—*p<0.05.

We created a ΔPDZ-MAS mutant that lacks the last five amino acids (containing the PBM) to validate MAS/PDZ protein interactions in cells. To evaluate the differences in WT and ΔPDZ-MAS mutant we established stable cell lines and studied activation of classical Gα_q_-phospholipase C signaling pathways by measuring the D-*myo*-inositol-1-phosphate (IP1) and calcium levels. The surface expression of ΔPDZ-MAS in the stable cell line was ~30% lower compared to the WT stable cell line (Fig 4A). The constitutive/basal IP1 levels in ΔPDZ-MAS were slightly lower but not statistically different compared to WT cell line (Fig 4B). The MAS-specific agonist (AR-agonist) and physiological ligand, neuropeptide FF (NPFF), increased IP1 levels in ΔPDZ-MAS stable cell line with an EC_50_ value which is not significantly different than the WT (Fig 4C). Similar to WT, ΔPDZ-MAS was also constitutively active and the MAS-specific inverse agonist (AR-inverse) inhibited the IP1 levels in a dose-dependent manner with an IC_50_ that is comparable to that of WT (Fig 4C). Next, we assayed the increase in intracellular calcium in ΔPDZ-MAS stable cell line in response to AR-agonist and NPFF in real-time as opposed to IP1 accumulation assay which is an end-point assay. In the calcium assays the kinetic parameter (t_1/2_) which is a measure of time taken to reach half of the maximal calcium response, was also measured along with dose-response curves. The EC_50_ of the AR-agonist and NPFF dose response was at least 2-fold higher and significantly different than that of WT (Fig 4D). The calcium flux kinetics (t_1/2_), upon treatment with AR-agonist was slower and significantly different compared to WT while the t_1/2_ observed upon NPFF treatment was faster and similar to the WT-response (Fig 4E). Our results suggest that the deletion of the PDZ-BM of MAS does not interfere with the constitutive activity of MAS and only moderately affects the activation of classical Gα_q_ signaling pathways. Thus, ΔPDZ-MAS mutant in terms of signaling is not very different from WT-MAS other than the lack of PDZ-BM which is expected to prevent MAS-PDZ protein interactions.

### Experimental validation of MAS Ct interacting proteins

**Fig 5:**
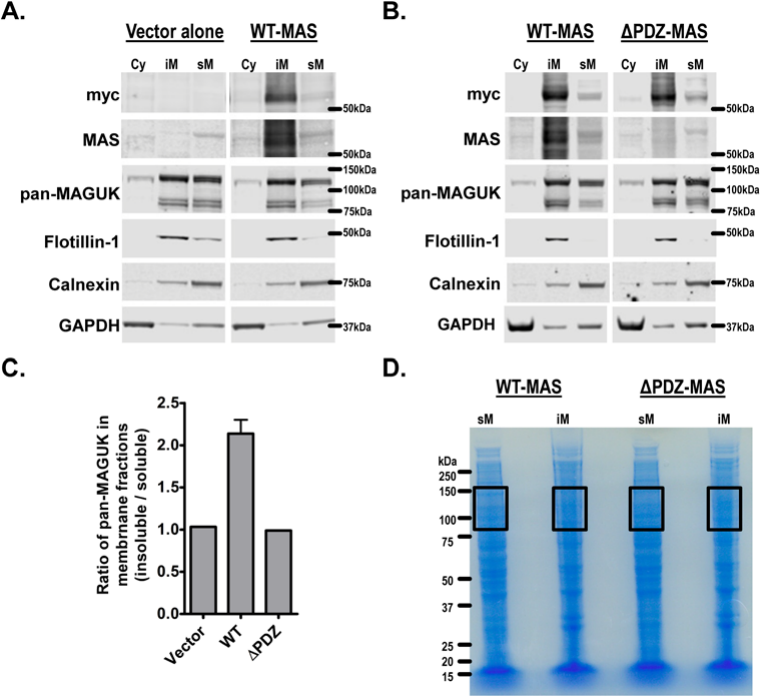
Interaction of MAS and PDZ proteins in HEK293 cells. Western blots of Cytoplasmic (Cy), detergent insoluble membrane (iM) and detergent soluble (sM) fractions from **(A)** Vector alone and WT-MAS and **(B)** WT-MAS and ΔPDZ-MAS transfected HEK293 cells. The western blots were initially probed with anti-c-myc antibody and then stripped and re-probed multiple times with different primary antibodies. The data for WT-MAS in panels (A) and (B) are from two independent experiments. **(C)** The pan-MAGUK signal in the western blots in panels (A) and (B) was as quantified using the Odyssey® Infrared Imaging System (LI-COR Biosciences, Lincoln, NE) and the ratio of pan-MAGUK band intensities of iM to sM fractions is shown. **(D)** The sM and iM fractions from WT-MAS and ΔPDZ-MAS were resolved on SDS-PAGE gels and the regions between ~75kDa to 150kDa (boxed regions) corresponding to the regions positive for pan-MAGUK signals (see panel B) were subjected for MS analysis to identify the PDZ proteins that were differentially enriched in different fractions.

We prepared cytosolic (Cy), detergent insoluble membrane (iM) and detergent soluble membrane (sM) fractions from transfected HEK293 cells with vector alone, myc-tagged wild-type (WT) MAS or ΔPDZ-MAS mutant. These Cy, iM and sM fraction preparations were enriched for respective marker proteins GAPDH, flotillin-1 and calnexin (Fig 5A and 5B). The WT-MAS was enriched in the iM fractions of cells as seen by the positive staining of anti-myc antibody and an antibody raised against the cytoplasmic region of MAS (datasheet from supplier). The ΔPDZ-MAS mutant was not detected by this antibody suggesting likely overlap of the antibody binding epitope region with that of PDZ-BM. However, the anti-myc antibody signal indicated that mutant lacking ΔPDZ-BM partitions predominantly into iM but a significant portion partitioned into sM fraction (Fig 5B). The pan-MAGUK antibody that was used to verify the presence of PDZ proteins in pull down assays (Fig 1C) detected PDZ proteins bands in the molecular weight range of 75 kDa to 150 kDa in the vector alone control, WT and ΔPDZ membrane fractions. There was a 2-fold increase in the pan-MAGUK signal in iM fraction compared to sM in the WT but not the ΔPDZ mutant (Fig 5C). This specific enrichment of PDZ proteins in the iM fractions of only WT suggests likely direct interaction of full-length MAS and PDZ proteins.

To further validate the protein hits from pull-down assays and to unambiguously identify the subset of MAS interacting PDZ proteins that are detected by pan-MAGUK antibody we performed MS analysis of specific regions in the sM and iM fractions from WT and ΔPDZ-MAS (Fig 5D). A total of 875 proteins were identified by MS in these regions (S4 Table). Of these 10 PDZ proteins were significant hits with spectral counts greater than 10 (Table 3 and S4 Table). The abundance of proteins in the iM compared to sM fractions and the specific enrichment of PDZ proteins in WT compared to ΔPDZ-MAS is provided in Table 3. The proteins DLG1 and TJP2 are >2-fold higher in the iM fractions of WT compared to ΔPDZ-MAS suggesting high affinity direct interactions of these proteins with MAS PDZ-BM. Other protein hits such as SCRIB, CSKP, TJP1 and MPP5 show no such enrichment and likely suggest low-affinity or lack of direct interactions of these proteins with MAS. Interestingly, the proteins NEB1, NEB2 and PDZD8 were absent in our pull-down assays and also show no differences in their abundance in WT and ΔPDZ membrane fractions, thus, serving as internal controls for our experimental validation approach. The observed increase (2-fold) in pan-MAGUK signal and the corresponding enrichment (also 2-fold) of DLG1 and TJP2 in the iM fractions of WT-MAS independently suggests direct binding and sequestration of specific PDZ proteins by MAS. Overall, these findings provide experimental validation of our proteomic approach.

**Table 3:** List of PDZ proteins identified to be significantly present in the membrane fractions of WT and ΔPDZ from areas highlighted by boxes on the protein gel in Fig 5B.

Uniprot name	WT (iM/sM)^†^	ΔPDZ (iM/sM)	Fold enrichment in WT*
**DLG1**	2.81	1.25	**2.2**
**TJP2**	4.9	2.27	**2.2**
**CSKP**	1.34	1.12	1.2
**SCRIB**	1.42	1.44	1.0
**TJP1**	43.15	45.65	0.9
**NEB2** ^‡^	26.48	28.06	0.9
**NEB1** ^‡^	only in iM^§^	only in iM	—
**MPP5**	only in iM	only in iM	—
**PDZD8**	only in sM^§§^	only in sM	—
**AFAD**	not significant^¶^	0.44	—

^†^Ratio of normalized spectral counts (nSC; see methods) of detergent insoluble (iM) over detergent soluble (sM) membrane fractions

^‡^Proteins not identified in the pull-down assays with MAS Ct

*Fold enrichment is the ratio of iM/sM of WT relative to ΔPDZ-MAS

^§^SC > 10 in the iM while the SC = 0 in sM fractions.

^§§^SC > 10 in sM while the SC = 0 in iM fractions. ^§§^SC > 10 in sM while the SC = 0 in iM fractions.

^¶^SC < 10 in both sM and iM fractions.

## Discussion

Understanding molecular mechanism of MAS function is paramount in developing novel therapeutics targeting MAS function. We recently demonstrated classical and atypical signaling and functional desensitization behavior of MAS which led us to hypothesize that G protein-independent receptor-scaffold protein interaction may modulate MAS signaling response [23]. In this study we set out to characterize the proteins interacting with MAS Ct. MAS Ct contains a PDZ-BM which is a well-defined protein-protein interaction motif. Using pull down and MS based proteomic approaches we identified several PDZ proteins to interact with MAS from three independent samples, HEK293 (human embryonic kidney cell line), HL-1 (mouse cardiomyocyte) and human cardiac tissue lysates. Relatively large numbers of PDZ protein hits were identified in HEK293 and HL-1 cell lysates. The cardiac tissue lysate yielded only 3 hits most likely due to the presence of highly differentiated cells in these tissues. Interestingly, the protein hits from cardiac lysates suggest the presence of a ‘cardiac-specific finger print’ containing the PDZ proteins DLG1, MAGI1 and SNTA. The PDZ-BM of TGFα precursor protein (non-GPCR) and Frizzled-4 (GPCR) are identical to MAS and these proteins were shown to interact with MAGI3 [49,50]. In another study, the Ct peptides containing PBZ-BM of Frizzled-4 and MAS were shown to bind to MAGI1 and scribble (SCRIB) [37]. The presence of MAGI3, MAGI1 and SCRIB in our hits indirectly validates our proteomic approach. Previously, PSD-95 (also known as DLG4 or SAP90) from the rabbit brain tissue lysate was shown to interact with MAS Ct [36]. PSD-95 was not present in any of our cell or tissue lysates highlighting cell-type dependent interactions of MAS and PDZ proteins might be at play.

Presence of non-PDZ proteins as significant hits suggests that we identified multi-protein complexes likely engaged by MAS Ct. PDZ proteins are primarily scaffolding proteins that interact with several proteins (including non-PDZ and other PDZ proteins) and assemble supramolecular protein complexes [29]. Previous studies using a similar experimental approach as ours have reported identification of similar multi-protein complexes of PDZ and non-PDZ proteins [51]. The presence of well-known signaling (DAPC) and scaffolding complexes regulating cell-cell junction assembly and stability (S1 Fig) in our hits provides insights regarding possible novel cellular functions of MAS.

We provide direct validation of MAS and PDZ protein interactions in cells using a pan-MAGUK antibody that identifies multiple PDZ proteins belonging to MAGUK sub-family and by estimating the protein enrichment in the corresponding regions by MS. There is clear enrichment of PDZ proteins along with MAS in membrane factions of cells expressing WT-MAS but not in ΔPDZ-MAS, MAS mutant that lacks the PBZ-BM. Interestingly, the lack of PDZ interactions in ΔPDZ-MAS has negligible effect on the receptor trafficking to the detergent insoluble membrane fractions. Further, there were no significant differences in the IP1 accumulation in the presence of agonist or inverse agonist treatments in ΔPDZ-MAS compared to WT-MAS. The decrease in IP1 levels in presence of inverse agonist also suggests that similar to WT, ΔPDZ-MAS also exhibited constitutive activity. However, compared to WT-MAS, agonist induced calcium signaling was moderately (2-fold) affected in ΔPDZ-MAS suggesting that PDZ proteins modulate MAS signaling. We speculate that the PDZ-MAS interactions might have a bigger role on downstream events following receptor activation such as receptor phosphorylation, internalization and degradation. For example, a MAS-GFP fusion protein with GFP at the C-terminus which would abolish PDZ interactions showed poor internalization compared to WT-MAS suggesting a possible role for PDZ protein interactions in internalization [52]. In a recent study, MAS interaction with PSD95 is shown to inhibit Mas receptor degradation via the ubiquitin-proteasome pathway [36].

To our knowledge this is a first report to comprehensively identify a panel of PDZ and non-PDZ proteins interacting either directly or indirectly with MAS from cell and human cardiac tissue lysates. Ingenuity pathway analysis of the hits suggests a multi-protein complex playing major role in cell-cell junction and nNOS signaling pathways. Also, the major disease categories involving MAS Ct interacting proteins were cancers and malignant tumors. These findings are not surprising as (1) MAS is reported to be involved in ischemic-reperfusion injury in heart which is an event primarily mediated by cell-cell junction signaling [20,21,53] and (2) MAS was discovered as an oncogene and several studies report transformation of cells overexpressing MAS including up-regulation of MAS in human colon adenocarcinoma [2–5,54,55]. Specific mutations in SNTA, which is one of the proteins in the proposed ‘cardiac-specific finger print’, are reported to cause long QT syndrome by altering its association with neuronal nitric oxide synthase (nNOS), cardiac sodium channel (SCN5A) and plasma membrane Ca-ATPase subtype 4b (PMCA4b) [56]. In skeletal muscles SNTA is known to play a key role in NO signaling by assembling multi-protein complexes [45,57]. Taken together we are tempted to propose possible role of SNTA in MAS mediated NO signaling.

**Fig 6:**
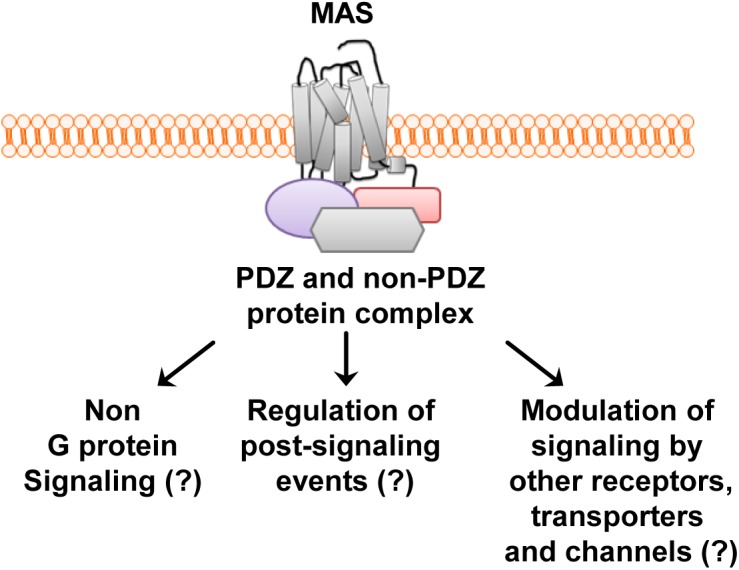
Hypothetical ‘MAS-signalosome’ model. The figure shows hypothetical assembly of PDZ and non-PDZ proteins leading to the formation of a signalosome. The ‘MAS-signalosome’ can potentially initiate novel signaling pathways or modulate different signaling or post-signaling events by MAS or other receptors.

Based on our findings and current understanding of GPCR-PDZ interactions we propose that PDZ proteins enable assembly of multiple protein complexes that likely mediate scaffold-dependent signaling pathways regulating different tissue-specific MAS function. Such ‘GPCR-signalosomes’ have been reported for metabotropic glutamate receptors, γ-aminobutyric acid type-B receptors, parathyroid hormone 1 receptor and α_1D_-adrenergic receptor (α_1D_-AR) [58–60]. Interestingly, major components of the α_1D_-AR/DAPC signalosome, SNTA1, SNTB1, SNTB2, CTNL1, DMD, DTNA, DTNB and UTRO, that sensitizes functional response of α_1D_-AR are recapitulated in our screen for a similar multiple protein assembly for MAS [58]. Our MS-based spectral counting quantitation provides strong evidence for existence of ‘MAS-signalosome’. A hypothetical model of a ‘MAS-signalosome’ is shown in Fig 6. The scaffold-mediated signaling pathways might act independently or synergistically with G protein signaling or modulation of post-signaling events such as receptor desensitization and degradation or recycling. The lack of independent co-immunoprecipitaion evidence for MAS association with each of interacting protein in the current study is a limitation for consolidating the arrangement and order of components in ‘MAS-signalosome’. Our future work is directed to validate and explore the spatio-temporal dynamics of signalosome assembly and its effect on MAS function.

## Supporting Information

S1 Fig Protein association network of MAS Ct interacting proteins. The protein interactions are identified using STRING database (http://string-db.org/). Human protein hits (HEK293 and cardiac tissue) were uploaded on the database and protein-protein interactions were predicted with a medium confidence score cut off (0.4). Proteins are represented by nodes while the edges denote the interaction. The thickness of edges is directly correlated to the confidence of interaction. Proteins which were not part of any interactions are removed from the figure to enhance clarity. The dystrophin-associated protein complex (DAPC) complex cluster involving PDZ proteins (SNTA1, SNTB1 and SNTB2) and non-PDZ proteins (DMD, DTNA, DTNB and UTRO) is highlighted by a red box. The clusters involving known tripartite complexes (MPP7-DLG1-LIN7; DLG1-CASK-LIN7) are highlighted by a green box. The TJP1 (also known as ZO-1), TJP2 (also known as ZO-2) and AFAD (also known as MLLT4) cluster is highlighted by a black box.
(TIF)

S1 Table MS data from the pull down assay with HEK293 lysates. List of all the proteins identified by MS in the elution fractions of resin alone and Ct bound resin from the pull down assay with HEK293 lysates. Data is provided as an excel file along with the spectral counts, normalized spectral ratios, protein accession numbers and additional annotations.
(XLSX)

S2 Table MS data from the pull down assay with HL-1 lysates. List of all the proteins identified by MS in the elution fractions of resin alone and Ct bound resin from the pull down assay with HL-1 lysates. Data is provided as an excel file along with the spectral counts, normalized spectral ratios, protein accession numbers and additional annotations.
(XLSX)

S3 Table MS data from the pull down assay with cardiac tissue lysates. List of all the proteins identified by MS in the elution fractions of resin alone and Ct bound resin from the pull down assay with cardiac tissue lysates. Data is provided as an excel file along with the spectral counts, normalized spectral ratios, protein accession numbers and additional annotations.
(XLSX)

S4 Table MS data for the validation experiments from WT and ΔPDZ-MAS expressing HEK293 cells. List of proteins in the 75kDa to 150kDa molecular weight range (areas marked on the protein gel in Fig 5D) identified by MS in the detergent resistant and detergent soluble fractions of membrane preparations from WT and ΔPDZ-MAS expressing HEK293 cells. Data is provided as an excel file along with the spectral counts, normalized spectral ratios, protein accession numbers and additional annotations.
(XLSX)
