# Supplementary figures and images for: MAS C-Terminal Tail Interacting Proteins Identified by Mass Spectrometry- Based Proteomic Approach
Source: PLoS One. 2015 Oct 20;10(10):e0140872. doi: 10.1371/journal.pone.0140872 (PMC4618059; doi:10.1371/journal.pone.0140872)

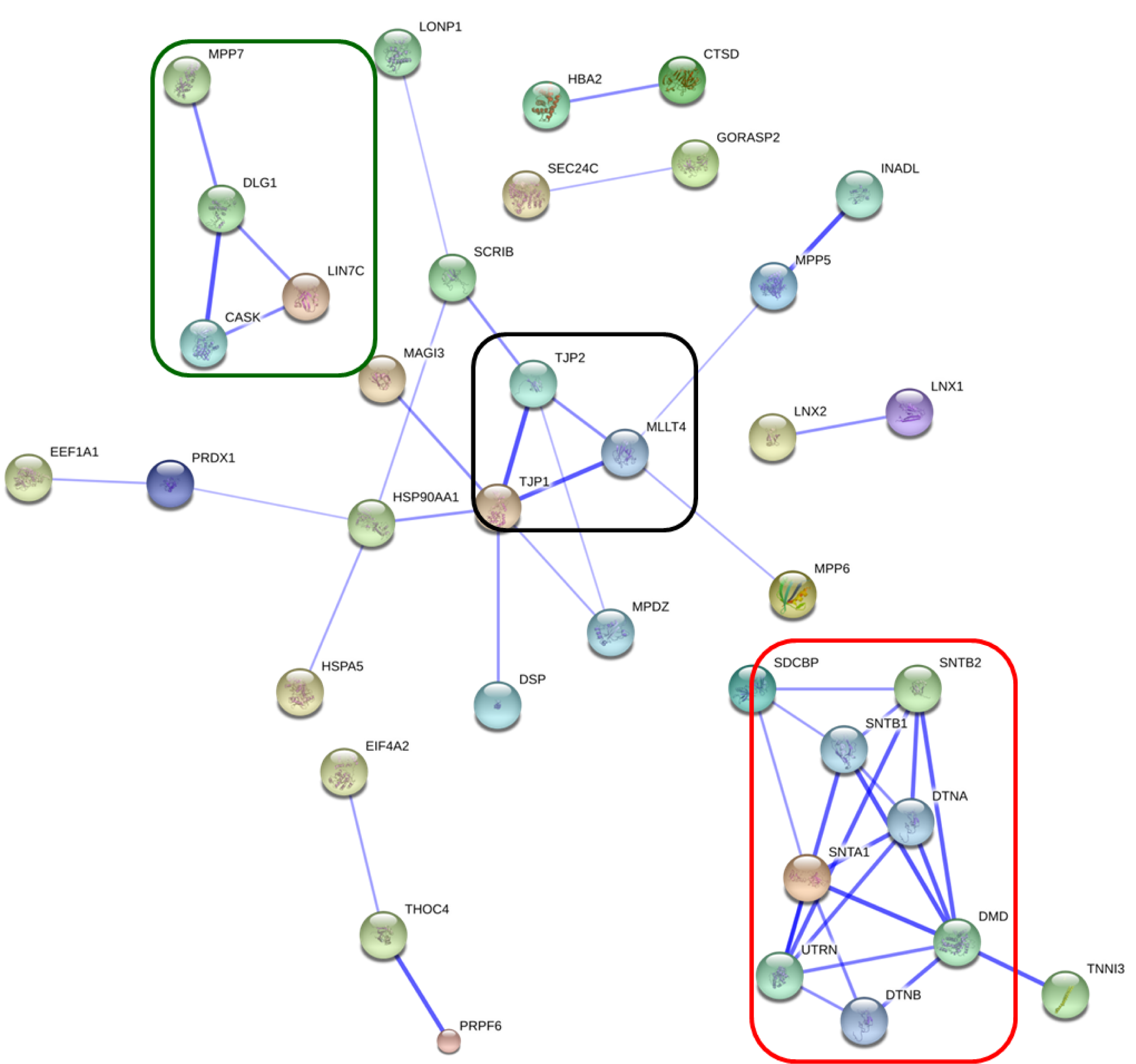

Supplement: S1 Fig — The protein interactions are identified using STRING database (http://string-db.org/). Human protein hits (HEK293 and cardiac tissue) were uploaded on the database and protein-protein interactions were predicted with a medium confidence score cut off (0.4). Proteins are represented by nodes while the edges denote the interaction. The thickness of edges is directly correlated to the confidence of interaction. Proteins which were not part of any interactions are removed from the figure to enhance clarity. The dystrophin-associated protein complex (DAPC) complex cluster involving PDZ proteins (SNTA1, SNTB1 and SNTB2) and non-PDZ proteins (DMD, DTNA, DTNB and UTRO) is highlighted by a red box. The clusters involving known tripartite complexes (MPP7-DLG1-LIN7; DLG1-CASK-LIN7) are highlighted by a green box. The TJP1 (also known as ZO-1), TJP2 (also known as ZO-2) and AFAD (also known as MLLT4) cluster is highlighted by a black box. (TIF) [file pone.0140872.s001.tif]
